# Supplementary material for: Genetic Characteristics and Long-Term Follow-Up of Slovenian Patients with RPGR Retinal Dystrophy
Source: Int J Mol Sci. 2023 Feb 14;24(4):3840. doi: 10.3390/ijms24043840 (PMC9958649; doi:10.3390/ijms24043840)
Supplement: Supplementary file 1 [file ijms-24-03840-s001.zip › ijms-2068455-supplementary.pdf]

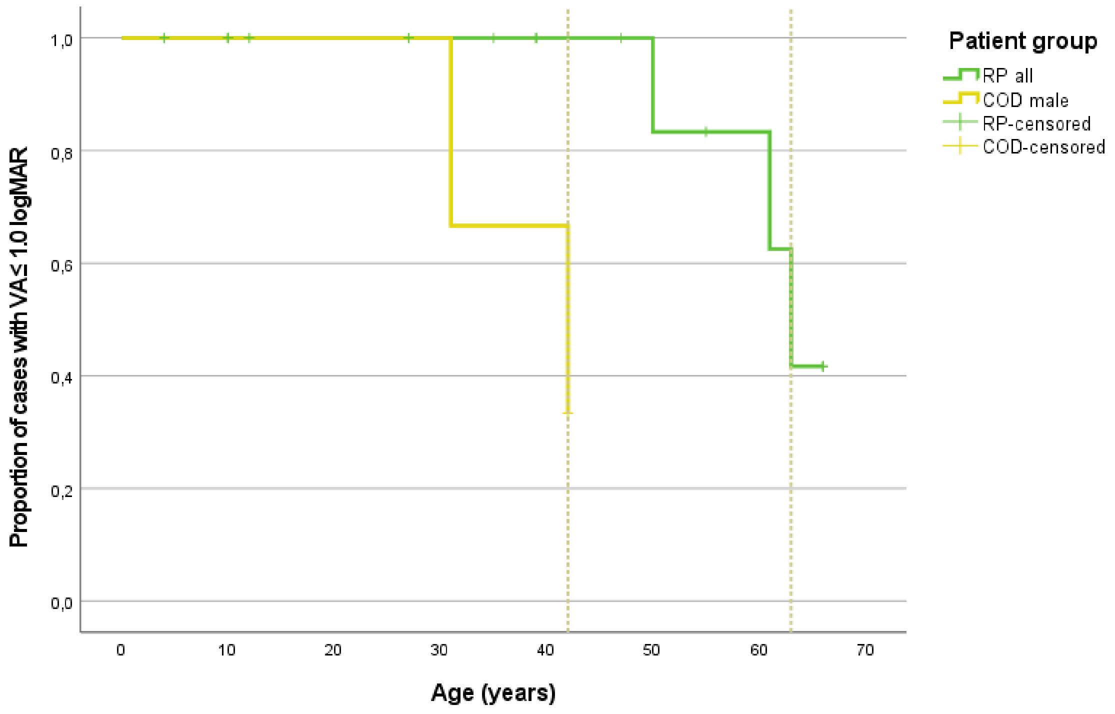

Figure S1. Kaplan-Meier Survival Analysis in all RP patients (including female carriers) and COD patients

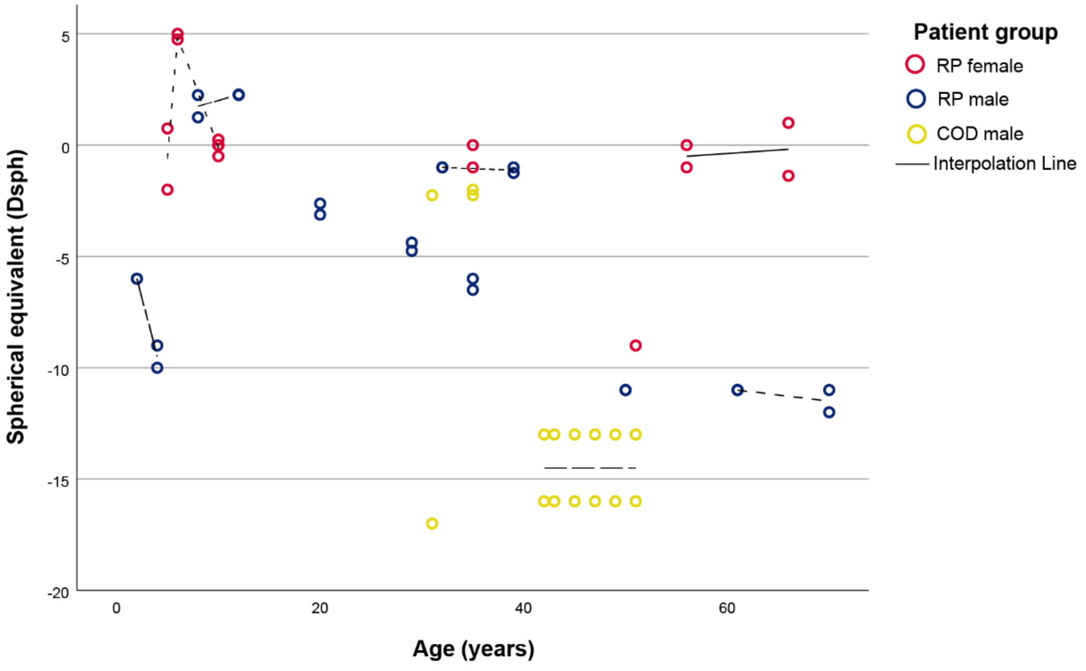

Figure S2. Spherical equivalent in relation to patients' age and diagnosis (both eyes are measured, showed both in the same vertical position related to age)

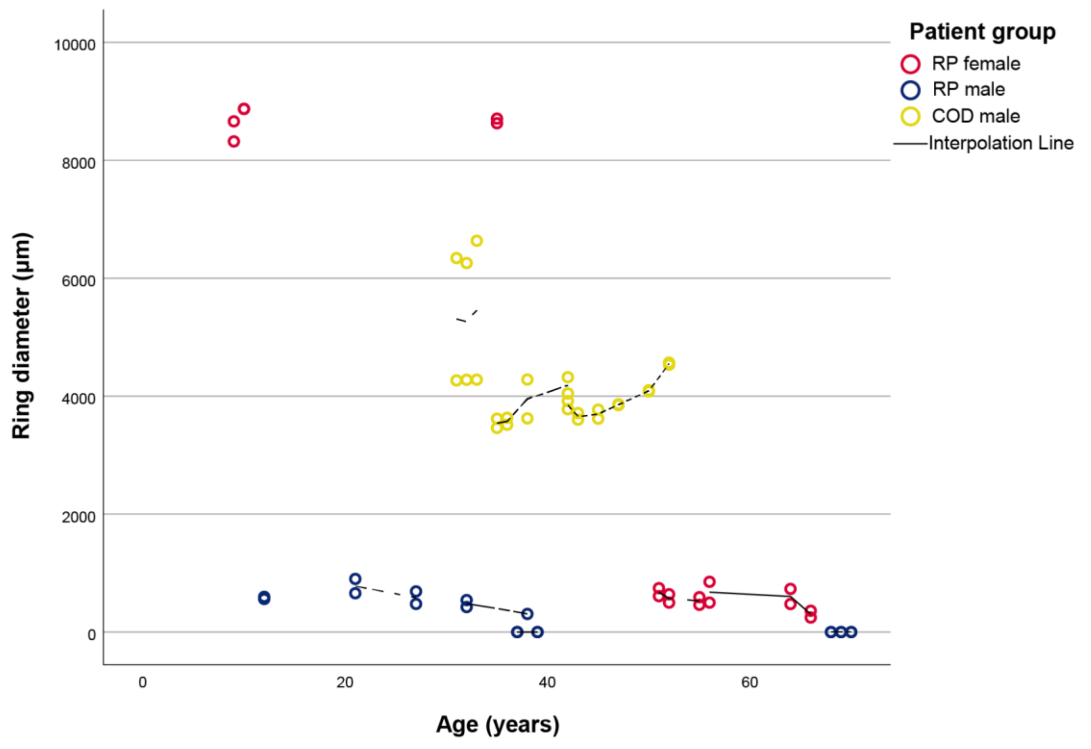

Figure S3. Ring diameter in relation to patients' age and diagnosis (both eyes are measured, showed both in the same vertical position related to age)

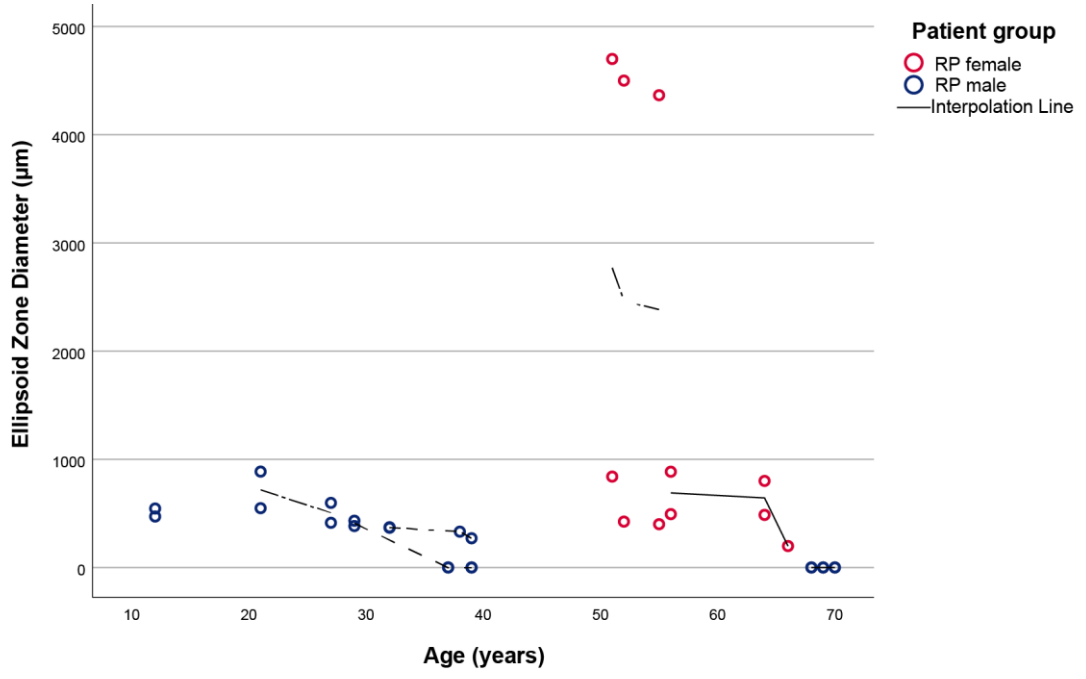

Figure S4. Ellipsoid Zone Diameter measured in patients with RP (both eyes are measured, showed both in the same vertical position related to age)

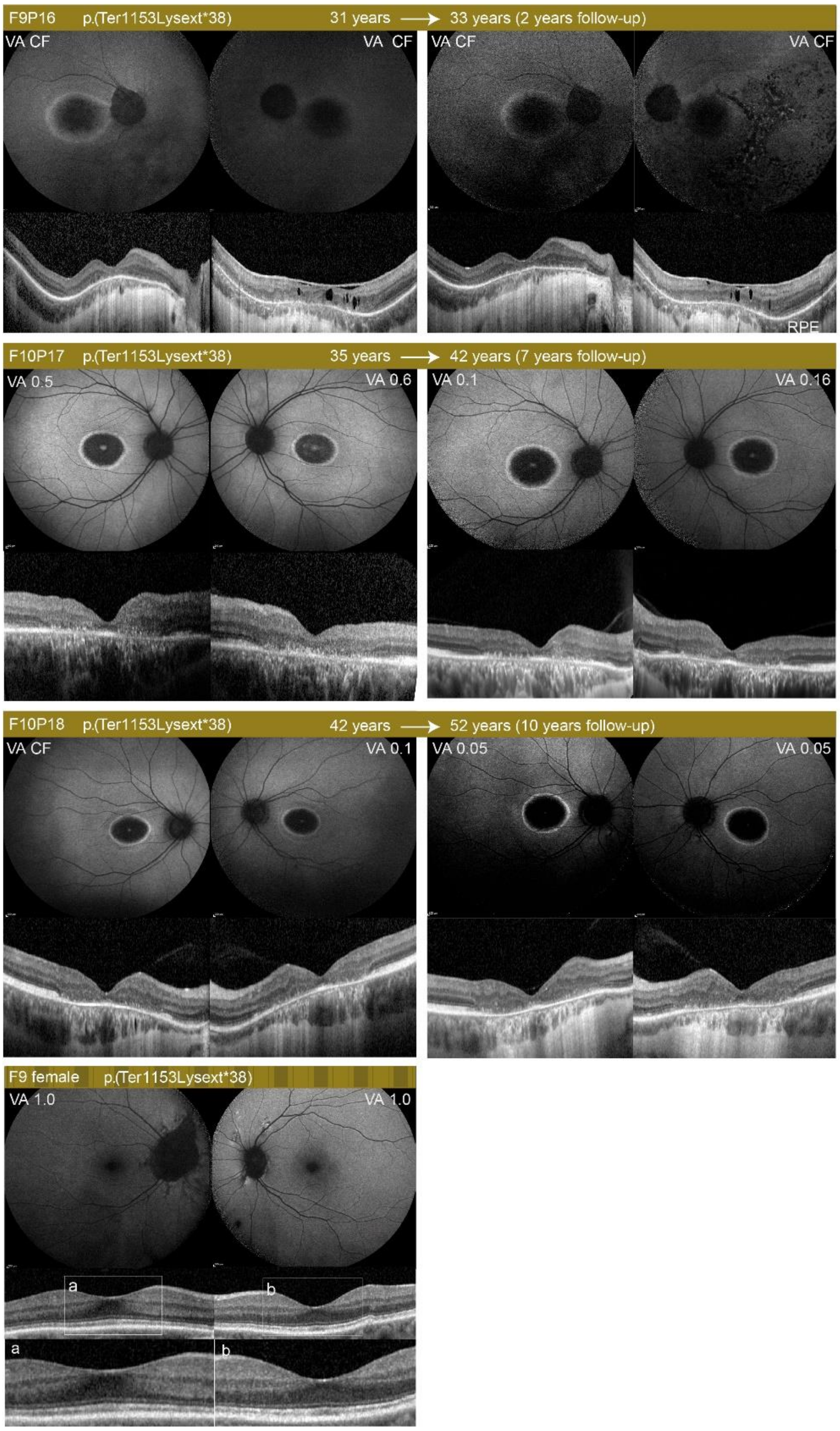

Figure S5. FAF and OCT of male and female carrier COD patients from families 9 and 10. Follow-up images are shown for each male patient on the right side of the image. OCT shows loss of photoreceptors inside of the hyperautofluorescent ring. Female carrier expressed normal FAF and OCT.
F-family, P-patient, VA-visual acuity, CF-counting fingers, RPE-retinal pigment epithelium.
